# Patient-Level Exposure to Actionable Pharmacogenomic Medications in a Nationally Representative Insurance Claims Database

**DOI:** 10.3390/jpm13111574

**Published:** 2023-11-03

**Authors:** Monica L. Bianchini, Christina L. Aquilante, David P. Kao, James L. Martin, Heather D. Anderson

**Affiliations:** 1 Skaggs School of Pharmacy and Pharmaceutical Sciences, University of Colorado Anschutz Medical Campus, Aurora, CO 80045, USA; monica.bianchini@cuanschutz.edu (M.L.B.); christina.aquilante@cuanschutz.edu (C.L.A.); james.3.martin@cuanschutz.edu (J.L.M.); 2 Colorado Center for Personalized Medicine, University of Colorado Anschutz Medical Campus, Aurora, CO 80045, USA; david.kao@cuanschutz.edu; 3 School of Medicine, University of Colorado Anschutz Medical Campus, Aurora, CO 80045, USA

**Keywords:** pharmacogenomic medications, medication exposure, pharmacogenomic testing, insurance claims, managed care

## Abstract

Background: The prevalence of exposure to pharmacogenomic medications is well established but little is known about how long patients are exposed to these medications. Aim: Our objective was to describe the amount of exposure to actionable pharmacogenomic medications using patient-level measures among a large nationally representative population using an insurance claims database. Methods: Our retrospective cohort study included adults (18+ years) from the IQVIA PharMetrics^®^ Plus for Academics claims database with incident fills of 72 Clinical Pharmacogenetics Implementation Consortium level A, A/B, or B medications from January 2012 through September 2018. Patient-level outcomes included the proportion of days covered (PDC), number of fills, and average days supplied per fill over a 12-month period. Results: Over 1 million fills of pharmacogenetic medications were identified for 605,355 unique patients. The mean PDC for all medications was 0.21 (SD 0.3), suggesting patients were exposed 21% (77 days) of the year. Medications with the highest PDC (0.55–0.89) included ivacaftor, tamoxifen, clopidogrel, HIV medications, transplant medications, and statins; with the exception of statins, these medications were initiated by fewer patients. Pharmacogenomic medications were filled an average of 2.8 times (SD 3.0, range 1–81) during the year following the medication’s initiation, and the average days supplied for each fill was 22.3 days (SD 22.4, range 1–180 days). Conclusion: Patient characteristics associated with more medication exposure were male sex, older age, and comorbid chronic conditions. Prescription fill data provide patient-level exposure metrics that can further our understanding of pharmacogenomic medication utilization and help inform opportunities for pharmacogenomic testing.

## 1. Introduction

Pharmacogenomics, a component of precision medicine, leverages patient-specific genetic information to inform medication prescribing with the goal of increasing effectiveness and decreasing adverse effects. Pharmacogenomic medications are medications for which there is an established genetic association that may impact therapeutic management. The US Food and Drug Administration has identified over 70 medications with data supporting therapeutic management recommendations or with data indicating a potential impact on drug safety or response [1]. The Clinical Pharmacogenetics Implementation Consortium (CPIC) assigns levels A through D to drug/gene pairs based on several factors, including clinical context, level of evidence, and strength of recommendation for prescribing actions. The strength of the recommendation increases from no prescribing action recommended (level D) to prescribing action recommended (levels A, A/B, and B). For example, CPIC has published 26 evidence-based guidelines for nearly 100 gene-drug pairs designated as CPIC level A, indicating the “preponderance of the evidence is high or moderate in favor of changing prescribing” [2,3,4,5]. Clinicians use CPIC guidelines to inform drug therapy decisions based on pharmacogenomic test results.

Several studies have evaluated populations most likely to benefit from pharmacogenomic testing [6,7,8,9,10,11]. One in particular concluded that patients with specific diagnoses, namely chronic kidney disease, diabetes, or a cardiac condition, should be targeted for pre-emptive testing in particular [10]. Otherwise, evidence of who should be tested is largely based on the prescribing prevalence of pharmacogenomic medications. A recent study estimated that 17,335 per 100,000 eligible adult patients in 11 US health systems were prescribed at least one CPIC level A medication in 2016, increasing from 15,719 per 100,000 adults in 2011 [8]. The same study found that over 7200 per 100,000 adult patients were prescribed at least two CPIC medications. Another study of nearly 75 million claims for adult patients with private insurance, Medicare supplement, or Medicaid found that about one-third to one-half of the patients were prescribed two or more pharmacogenomic medications from 2009–2012. Although there is evidence that pharmacogenomic testing has grown and the prescribing of medications with pharmacogenomic guidelines recommending changes in therapeutic management is common, questions remain about which patients to test, optimal testing strategies, and the value of pharmacogenomic testing in these populations [12,13,14,15].

While prescribing prevalence, or the number of patients prescribed a medication, has been the key metric used in pharmacogenomic value and clinical utility assessments, it primarily describes provider behavior and does not quantify patient-level exposure to medications. Patient-level metrics that use medication fill data, including the proportion of days covered (PDC), days supplied, number of fills, and total duration of exposure, provide a better picture of the amount of patient exposure to relevant medications in real-world scenarios. An understanding of the amount of exposure to pharmacogenomic medications can inform different aspects of pharmacogenomic implementation efforts, including testing, intervention, and evaluation. To our knowledge, no studies have comprehensively evaluated the amount of patient-level exposure to actionable pharmacogenomic medications in large patient cohorts. The objective of this study was to describe the amount of exposure to newly prescribed actionable pharmacogenomic medications using paid insurance claims data and patient-level fill measures among a large, nationally representative population.

## 2. Materials and Methods

### 2.1. Data Source and Setting

This study utilized IQVIA PharMetrics^®^ Plus for Academics, a longitudinal insurance claims database that includes fully adjudicated medical and pharmacy claims for over 100 million unique enrollees across the U.S. The University of Colorado Skaggs School of Pharmacy and Pharmaceutical Sciences holds a license for a random sample of approximately 11 million patients from PharMetrics^®^ Plus. Available claims-level variables include medications filled (medication code, fill date, and days supplied), diagnoses, procedures, and service dates. Patient-level demographic variables include year of birth, sex, region of residence, and type of insurance. Race and ethnicity information is not available. The enrollees included are nationally representative in terms of age and sex distributions of patients in the U.S. commercially insured population [16]. This study was approved as exempt human subjects research by the Colorado Multiple Institutional Review Board.

### 2.2. Study Design and Population

This was a retrospective cohort study of adults aged 18 years and older from the PharMetrics^®^ Plus database with an incident fill of one or more CPIC level A, A/B, or B pharmacogenomic medications from 1 January 2012 to 30 September 2018, with data from 1 October 2018 through 30 September 2019 used for follow-up. We include CPIC level A, A/B, and B medications because some level of prescribing action is recommended for these levels [4]. Specific medications were identified for study inclusion based on CPIC guidelines as of January 2022. Statins were added after receiving a level A recommendation in February 2022 [17]. The CPIC guideline for medications associated with glucose-6-phosphate dehydrogenase (G6PD) genotype was updated during this work, so G6PD-related medications expected to be classified as ‘low-to-no risk’ were excluded from this study. All CPIC level A, A/B, and B medications included can be found in Table S1. This study included 72 pharmacogenomic medications: 48 (66.7%) CPIC level A, 7 (9.7%) CPIC level A/B, and 17 (23.6%) CPIC level B.

Incident fills of pharmacogenomic medications were identified using generic product identifier medication codes specific to each medication and were defined as those with at least six months of continuous health plan enrollment prior to the fill with no evidence of the same medication filled during that time. An incident fill was excluded if there were less than twelve months of continuous health plan enrollment following the fill. Claims with zero or negative days supplied were excluded. Patients could be included in the study multiple times, once for each pharmacogenomic medication they initiated during the study period. The index date for each medication initiated was assigned as the earliest fill of that medication that met inclusion criteria.

### 2.3. Measures

#### 2.3.1. Amount of Exposure to Pharmacogenomic Medications

**Figure 1:**
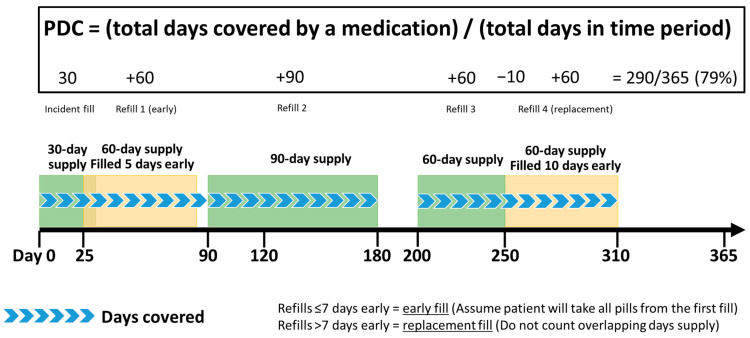
A graphic depiction of how the proportion of days covered (PDC) is calculated in this study, accounting for early and replacement medication fills.

The primary measure of patient-level exposure to each CPIC level A, A/B, or B pharmacogenomic medication was the PDC during the first year of use. The PDC is calculated by dividing the total days covered by a medication by the number of days in the observation period, accounting for overlapping fills (Figure 1). [18] For example, the PDC calculation for a patient who was covered by medication for 290 days in one year (365 days) would be equal to (290/365) = 0.79. This PDC indicates a patient was exposed to the medication for 79% of the days within the given time period. For this study, the total days covered were the total days supplied for all fills during the 12 months following the index fill minus any overlapping days supplied greater than seven days. Overlapping days supplied less than or equal to seven days were considered an early fill and it was assumed the patient still had medication from the current fill in-hand. Overlapping days supplied greater than seven days were assumed to be replacement fills and therefore overlapping days were not counted as days covered. Including overlapping days supplied that may be replacement fills would overestimate the PDC. Fills that overlapped the index date plus 365 days were truncated. The observation period and denominator were 12 months for all fills.

Other measures of patient-level medication exposure included the total number of fills of each pharmacogenomic medication and the mean days supplied for each fill across all fills of a medication, both measured during the first year of use after initiation.

#### 2.3.2. Clinical and Demographic Measures

In order to examine the patient-level comorbidity burden, the Charlson Comorbidity Index (CCI) score was measured for each pharmacogenomic medication a patient-initiated. Patients could therefore have had multiple CCI scores. The CCI score was calculated as a weighted sum of 17 unique chronic conditions for which the patient had a diagnosis code during the 6 to 12 months prior to the pharmacogenomic medication initiation, depending on the number of months of continuous enrollment [19]. Indicators for each of the 17 chronic conditions were also created so the prevalence of the unique diagnoses could be described.

Demographic characteristics included age in years, sex, region of residence (South, West, East, Midwest), and insurance type (commercial, managed Medicaid/Medicare, or other/unknown). Managed Medicaid/Medicare includes Medicaid or Medicare supplemental plans through commercial insurance providers. Demographic characteristics were measured at the patient level at the time of the earliest incident pharmacogenomic medication fill.

### 2.4. Missing Data

For each exposure variable and covariate, we defined outliers as values greater than or equal to two times the 99th percentile for that variable [20]. Incident pharmacogenomic medication fills with outlier days supplied were excluded. Outliers for all other variables were treated as missing.

### 2.5. Statistical Analysis

Continuous variables were described using mean, standard deviation (SD), and minimum and maximum. The median and interquartile range (IQR, 25th percentile–75th percentile) were reported for skewed continuous variables. Categorical variables were described using counts and percentages. Primary results were reported for each CPIC level because while each level recommends prescribing action, the three levels have varying degrees of evidence. Additional comparisons were made by stratifying the cohort based on age and sex; an ANOVA was used to compare the mean PDC across age groups, and *t*-tests were used to compare the mean PDC between males and females overall and within each age group. No statistical comparisons were made between CPIC levels since individuals could be represented in more than one of the CPIC-level groups. We estimated the correlation between the average PDC and the number of incident users across all medications using the Pearson correlation coefficient. All data analyses were performed using SAS 9.1 (SAS Institute Inc., Cary, NC, USA).

## 3. Results

### 3.1. Cohort Characteristics

We identified 1,035,918 incident fills of pharmacogenomic medications representing 605,355 unique patients from 1 January 2012 to 30 September 2018; 80.6% of patients initiated a CPIC level A medication, 3.5% initiated a CPIC level A/B medication, and 42.8% initiated a CPIC level B medication. Note that these groups are not mutually exclusive. Table 1 describes the characteristics of unique patients at the time of their earliest medication initiation in the dataset, overall, and for each CPIC level group. The average age was 45 years old and 59.7% of patients were female (n = 361,569). Most patients had commercial insurance (n = 456,052, 75.3%). The mean CCI score was 0.6 (SD 1.3), indicating patients had, on average, zero to one of the chronic conditions from the index during the 12 months prior to initiating a pharmacogenomic medication. Almost half of the patients in the study cohort (42.5%) initiated more than one pharmacogenomic medication (mean 1.7, SD 1.1; range 1–18) during the study period.

### 3.2. Amount of Exposure to Pharmacogenomic Medications

Patient-level measures of exposure to pharmacogenomic medications are described in Table 2. The average PDC for all initiated pharmacogenomic medications was 0.21 (SD 0.3, range 0.003–1.0), meaning patients were exposed to the pharmacogenomic medication, on average, for 21% of the year (i.e., 77 days) following drug initiation. CPIC level A medications, which accounted for 70.5% of incident fills, had an average PDC of 0.24 (SD 0.3, range 0.003–1.0). The medications with the highest PDCs among CPIC level A medications were ivacaftor (mean PDC = 0.89), tamoxifen (mean PDC = 0.67), and clopidogrel (mean PDC = 0.65) (Table S1). CPIC level A/B medications, which accounted for 2.1% of incident fills, had an average PDC of 0.44 (SD 0.3, range 0.003–1.0). These medications included tetrabenazine (mean PDC = 0.62), venlafaxine (mean PDC = 0.46), and hydralazine (mean PDC = 0.46) (Table S1). CPIC level B medications, which accounted for 27.4% of incident fills, had the lowest average PDC of 0.10 (SD 0.2, range 0.003–1.0). Although CPIC level B medications had the lowest average PDC overall, some specific CPIC level B medications had relatively high PDCs, including mycophenolic acid (mean PDC = 0.56), sertraline (mean PDC = 0.44), and methadone (mean PDC = 0.43) (Table S1). Of the 10 medications with the highest PDCs (0.56 to 0.89), all but one (tetrabenazine) were CPIC level A, encompassing a variety of drug classes and genes (Table S1).

**Figure 2:**
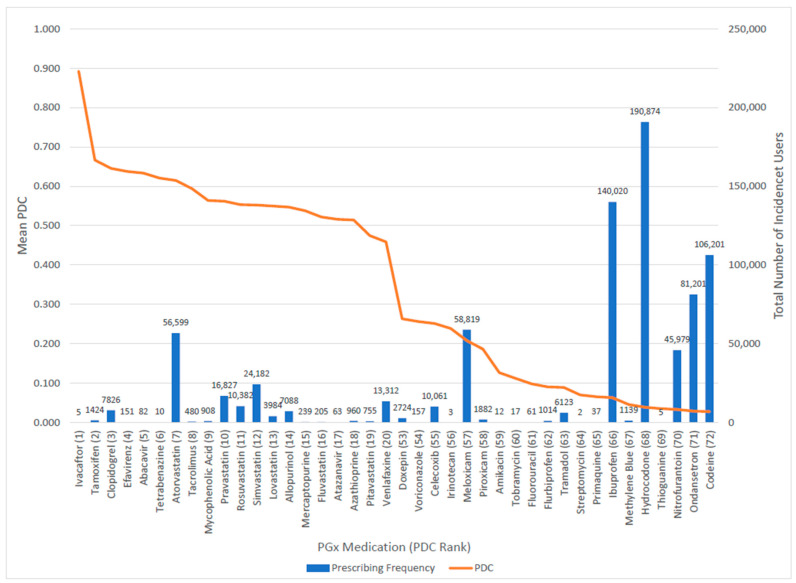
Mean proportion of days covered (PDC) (left vertical axis) and prescription fill frequency (i.e., number of incident fills; right vertical axis) for pharmacogenomic medications with the highest PDC (1–20) and lowest PDC (53–72).

We estimated a negative correlation between the average PDC and the number of patients with an incident fill across all medications included in the study (correlation coefficient = −0.343, *p* = 0.0032). This indicates that medications with higher mean PDCs tended to have fewer patients initiating the medication. This is evident in Figure 2, which shows the mean PDC (left vertical axis) decreased as the total number of patients with an incident fill (right vertical axis) increased for all 72 medications included in the study. Analgesic and anti-emetic medications (e.g., hydrocodone, ibuprofen, codeine, and ondansetron, in order of incident fill frequency) were among the most frequently filled medications but had the lowest PDC values ranging from 0.03–0.06 (average annual exposure of 11–22 days), consistent with medication used on a short-term basis. Generally, medications with the highest PDC values had fewer incident fills (e.g., ivacaftor, efavirenz, abacavir, and tetrabenazine), with statins as the major exception. For example, atorvastatin had the 7th highest PDC (0.62) and the seventh most incident users (n = 56,599) in the entire cohort.

Pharmacogenomic medications were filled, on average, 2.8 times (SD 3.0, range 1–81) during the year following the medication’s initiation, and the average days supplied for each fill was 22.3 days (SD 22.4, range 1–180) (Table 2). Five medications (ivacaftor, abacavir, tetrabenazine, tacrolimus, and efavirenz; PDCs = 0.59–0.89), all CPIC level A or A/B, were each filled, on average, more than seven times within the first year of use. Each fill represents a potential opportunity to make pharmacogenomic-guided changes to treatment. Tamoxifen and statins had the highest average days supplied, meaning patients went longer between fills. The average day supplied for tamoxifen in the first year of use was 51.4 days (SD 26.8). The average day supplied for statins ranged from 52.4 days (SD 26.5) for atorvastatin to 43.8 days (SD 22.9) for pitavastatin.

### 3.3. Medication Exposure by Patient Characteristics

Medication exposure varied based on patient characteristics, including age, sex, and comorbidities. Adults aged 80 and older had the highest average PDC (mean = 0.42, SD = 0.4) for pharmacogenomic medications, and the PDC decreased with decreasing age (*p* < 0.001). (Table 3). Overall, men had a higher PDC for pharmacogenomic medications (mean 0.23, SD 0.3) compared to women (mean 0.18, SD 0.3; *p* < 0.001), and the average PDC for men was the same or higher than women in each age strata (*p* < 0.01 to *p* = 0.003). Table 4 describes the average PDC across all pharmacogenomic medications for patients with comorbidities from the CCI score during the 12 months prior to the initiation of any pharmacogenomic medication during the study period. Patients with a diagnosis of a myocardial infarction had the highest PDC (mean 0.41, SD 0.3), while patients with chronic pulmonary disease had the lowest PDC (mean 0.23, SD 0.3). Patients with at least one diagnosis of the CCI had an average PDC of 0.27 (SD 0.32) compared to 0.17 (SD 0.28) for patients without any comorbidities (*p* < 0.001).

## 4. Discussion

This was a large U.S. cohort of over one million incident fills of actionable pharmacogenomic medications among 605,355 insured adults. Almost half of the patients (42.5%) initiated more than one pharmacogenomic medication during the study period, indicating patients were exposed to multiple pharmacogenomic medications over time. Patients were exposed to a pharmacogenomic medication, on average, for 21% of the year following drug initiation and filled the medication, on average, three times in that year. Importantly, these findings were largely driven by CPIC level A medications, which accounted for almost three-quarters of incident fills and represent drugs with moderate to high evidence in favor of changing prescribing.

The pharmacogenomic medications we identified as being filled by most patients were similar to those identified by prior studies as being most frequently prescribed (e.g., analgesics, antiemetics, antibiotics, and statins) [6,7,8,21]. We found that on average, patients were exposed to two pharmacogenomic medications during our study period. This is similar to a study of 7.7 million patients conducted in the VA population using pharmacy dispensing records, in which an estimated 37% of patients were exposed to at least one CPIC level A medication over 6 years and 25% were exposed to two CPIC level A medications. [7] Hicks et al., a study of 7.2 million adults, estimated the annual prevalence of adults being prescribed one CPIC level A medication to range from 15.7% to 17.3% and the annual prevalence of adults being prescribed at least two CPIC level A medications to range from 7.2% to 7.7% [8]. Similar to our study, several studies have described that the prevalence of pharmacogenomic medication prescribing increases with age [6,22,23]. It is worth noting that the highest PDC in this study was seen for ivacaftor, which had only five incident users. Ivacaftor is a unique pharmacogenomic medication because it is indicated for patients with certain *CTFR* mutations, specifically targeting gene defects associated with cystic fibrosis. Given the pathophysiology of the disease and limited treatment options, patients remain on the medication long-term; thus, exposure is expected to be high.

The results of this study can be used to inform population- or system-level implementation efforts. For example, preemptive or reactive pharmacogenetic testing would likely be advantageous for patients anticipated to be on pharmacogenetic medications with high PDCs. These patients have greater long-term drug exposure and repeated fills, thus offering more opportunities for genotype-guided interventions, depending on the medication. From a frequency perspective, these medications are prescribed to fewer patients; therefore, the implementation approach may be narrower in scope (e.g., select clinics) for certain agents. In contrast, patients anticipated to be on medications with low PDCs have less exposure to the drug and infrequent repeat fills. Preemptive testing is likely to be the preferred testing approach in these scenarios as prescribers are not likely to wait on pharmacogenomic test results to make clinical decisions, and there are fewer opportunities to make prescribing changes after the medication is initially filled. At the same time, these medications are prescribed with the highest frequency; therefore, a broader implementation approach (e.g., system-wide) has the potential to impact the greatest number of patients. In general, higher PDC estimates for medications indicate patients are exposed to those medications for larger portions of the year following initiation and could be at risk for suboptimal therapy or side effects over longer periods of time if pharmacogenomic results were not considered in the medication prescribing process. This is particularly true for CPIC level A medications, which have moderate to high evidence supporting prescribing changes.

Understanding the amount and timing of exposure to pharmacogenomic medications, rather than just the frequency of prescribing, can also inform the design of CDS tools for delivering pharmacogenomic-based prescribing recommendations. For many pharmacogenomic medications, a prescription fill represents an opportunity to make prescribing changes if alterations have not already been made with the incident fill or if the patient’s clinical scenario changes. If a medication is rarely filled, interruptive or asynchronous CDS tools may be necessary, depending on the anticipated severity of the adverse outcome. Along the same lines, if patients receive larger days supplied, meaning they do not need to fill the medication as often (e.g., statins), CDS tools that effectively strike the balance between notifying clinicians of the drug-gene interaction in a timely manner while also minimizing alert fatigue and cognitive load should be considered. For pharmacogenomic medications with high fill frequencies and low PDCs, passive tools may be most desirable for medications with mild to moderate adverse effects, while interruptive tools may be reserved for high-risk scenarios.

A limitation of this study is that pharmacy claims do not capture the majority of pharmacogenomic testing so we did not account for the receipt of pharmacogenomic testing, which could influence the timing and amount of exposure to pharmacogenomic medications. The claims database used does not include race or ethnicity, which would be useful for calculating the prevalence of actionable drug-gene pairs in the cohort. We do not have access to inpatient medication claims, so medications administered during a hospital stay are not included. Finally, CPIC guidelines and level assignments are subject to change—the medications included in this study and their corresponding CPIC level assignment were current as of January 2022.

There are other study limitations, including the retrospective study design and use of insurance claims data. Medications filled include only those filled through insurance and do not include medications filled out-of-pocket or out-of-network. While not a direct measure of whether patients take the medications they fill, claims data are frequently used to measure medication exposure and are preferred over other data sources like pharmacy dispensing records [24,25,26]. We did not measure exposure to a therapeutic class or account for medication changes within the class (e.g., statins). For example, if a patient were newly initiated on simvastatin and filled the medication for three months before switching to rosuvastatin, this patient would have been associated with two incident fills despite the fact that they were for the same medication class. A limitation related to our study design is that patients must have survived at least 12 months after filling their index pharmacogenomic medication in order to be included and therefore represent a healthier cohort. Recommendations for future research include the use of a prospective study design and the inclusion of other measures, such as the receipt of pharmacogenomic testing.

## 5. Conclusions

To our knowledge, this is the first study to quantify the amount of exposure to actionable pharmacogenomic medications using patient-level measures using fill data rather than prescription data, which reflects clinicians’ prescribing behavior rather than patient-level exposure to medications [7,8,9,10,11]. In this nationally representative insurance claims cohort, participants were exposed to pharmacogenomic medications for over 20% of the year after medication initiation, with multiple refills over the year. Exposure to CPIC level A medications, which have moderate to high evidence supporting prescribing changes, was even higher, with a mean PDC of 24% and an average of two refills (three total fills). Ultimately, these patient-level measures of exposure to pharmacogenomic medications with moderate to high evidence for prescribing changes have the potential to impact patient- and community-level health care by informing the design of CDS tools for pharmacogenomic testing implementation and characterizing opportunities for pharmacogenomic testing in different populations.

## Figures and Tables

**Table 1:** Characteristics of unique patients who filled at least one CPIC level A, A/B, or B pharmacogenomic medication during the study period (N = number of unique patients within each group ^1^).

Demographics	Total Cohort ^1^ (N = 605,355)	CPIC Level A (N = 487,800)	CPIC Level A/B (N = 21,059)	CPIC Level B (N = 258,994)
Age, mean (SD, **min–max**)	45.1 (15.2, 18–83)	45.7 (15.1, 18–83)	45.6 (15.0, 18–83)	43.5 (15.3, 18–83)
Female sex, n (%)	361,569 (59.7)	291,211 (59.7)	14,065 (66.8)	162,038 (62.6)
Region ^2^				
South, n (%) West, n (%) East, n (%) Midwest, n (%)	154,575 (25.5)	123,622 (25.3)	4700 (22.3)	67,586 (26.1)
	146,698 (24.2)	118,806 (24.4)	5024 (23.9)	66,671 (25.7)
	140,823 (23.3)	116,162 (23.8)	4610 (22.0)	51,676 (20.0)
	139,532 (23.1)	109,900 (22.5)	5853 (27.8)	62,445 (24.1)
Insurance Type				
Commercial, n (%) Managed Medicaid/Medicare, n (%) Other, n (%)	456,052 (75.3)	360,156 (73.8)	13,650 (64.8)	194,944 (75.3)
	142,250 (23.5)	121,436 (24.9)	7073 (33.6)	60,544 (23.4)
	7053 (1.2)	6208 (1.3)	336 (1.6)	3506 (1.4)
Charlson Comorbidity Index, mean (SD, min-max) ^3^	0.6 (1.3, 0–21)	0.6 (1.4, 0–21)	1.1 (1.9, 0–21)	0.6 (1.3, 0–21)
Total pharmacogenomic medications initiated ^4^, mean (SD, min-max)	1.7 (1.1, 1–18)	1.9 (1.4, 1–18)	2.9 (1.8, 1–16)	2.1 (1.3, 1–18)

^1^ Patients can be represented once per each CPIC level but are only included in the total cohort column once. Total cohort N is thus less than the sum of Ns for the three CPIC levels. ^2^ Frequency missing: total cohort = 23,727 (3.9%); CPIC Level A = 19,310 (4.0%); CPIC Level A/B = 872 (4.1%); CPIC Level B = 10,616 (4.1%). ^3^ Patients could have more than one Charlson Comorbidity Index measure (one for each pharmacogenomic medication initiated during the study period); the mean was calculated by including all measures for each patient. ^4^ CPIC level A, A/B, and B medications initiated during the 6-year study period.

**Table 2:** Amount of exposure to CPIC level A, A/B, or B pharmacogenomic medications by CPIC level.

	Total Cohort (N = 1,035,918) ^1^	CPIC Level A (N = 730,736) ^1^	CPIC Level A/B (N = 21,663) ^1^	CPIC Level B (N = 283,519) ^1^
	Mean (SD, min–max) Median (IQR)	Mean (SD, min–max) Median (IQR)	Mean (SD, min–max) Median (IQR)	Mean (SD, min–max) Median (IQR)
Proportion of days covered ^2^	0.21 (0.3, 0.003–1.0) 0.07 (0.02–0.25)	0.24 (0.3, 0.003–1.0) 0.08 (0.02–0.35)	0.44 (0.3, 0.003–1.0) 0.33 (0.08–0.79)	0.10 (0.2, 0.003–1.0) 0.02 (0.01–0.05)
Number of fills ^2^	2.8 (3.0, 1–81) 1 (1–3)	3.0 (3.1, 1–81) 1 (1–4)	5.1 (4.2, 1–47) 4 (1–8)	2.1 (2.5, 1–45) 1 (1–2)
Average days supplied across all fills ^2^	22.3 (22.3, 1–180) 15 (5–30)	26.2 (23.5, 1–180) 30 (7–30)	36.0 (17.1, 1–100) 30 (30–30)	11.2 (14.3, 1–120) 5 (3–10)

^1^ N = total number of incident fills in each group. ^2^ Each measure of amount of exposure was measured during the one year following the initiation of the pharmacogenomic medication.

**Table 3:** The average proportion of days covered (PDC) for CPIC level A, A/B, or B medications, by age and sex ^1^.

Age Group ^1^	N (%)	Unique Patients (N = 605,355) ^2^	Males (N = 243,764) ^1^	Females (N = 361,569) ^1^
		PDC, Mean (SD)	PDC, Mean (SD)	PDC, Mean (SD)
18–29	120,432 (19.9)	0.10 (0.19)	0.10 (0.20)	0.10 (0.19)
30–39	106,302 (17.6)	0.14 (0.24)	0.15 (0.25)	0.13 (0.22)
40–49	119,793 (19.8)	0.19 (0.28)	0.21 (0.30)	0.18 (0.27)
50–59	146,903 (24.3)	0.25 (0.32)	0.27 (0.34)	0.23 (0.31)
60–69	86,954 (14.4)	0.30 (0.35)	0.32 (0.36)	0.28 (0.34)
70–79	23,101 (3.8)	0.37 (0.38)	0.39 (0.39)	0.35 (0.37)
80+	1870 (0.3)	0.42 (0.40)	0.46 (0.40)	0.40 (0.39)

^1^ Statistically significant differences for mean PDC across age groups (*p* < 0.001) and between sexes overall (*p* < 0.001) and within each age group (*p* < 0.001). ^2^ N = 22, with missing data for sex.

**Table 4:** The average proportion of days covered (PDC) among patients with a diagnosis included in the Charlson Comorbidity Index (N = 605,355).

Diagnoses from the Charlson Comorbidity Index	N (%)	PDC, Mean (SD, Min–Max)
No Charlson Comorbidity Index diagnoses	428,691 (70.8)	0.17 (0.28, 0.003–1.0) ^1^
≥1 Charlson Comorbidity Index diagnoses	176,664 (29.2)	0.27 (0.33, 0.003–1.0) ^1^
**Specific Diagnoses**		
Myocardial Infarction	6933 (1.2)	0.41 (0.3, 0.003–1.0)
Diabetes without complications	69,993 (12.2)	0.31 (0.3, 0.003–1.0)
Diabetes with complications	15,486 (2.7)	0.33 (0.3, 0.003–1.0)
Paraplegia and Hemiplegia	2607 (0.5)	0.33 (0.3, 0.003–1.0)
Renal Disease	11,201 (2.0)	0.34 (0.3, 0.003–1.0)
Cancer	21,821 (3.8)	0.26 (0.3, 0.003–1.0)
Moderate or Severe Liver Disease	1029 (0.2)	0.28 (0.3, 0.003–1.0)
Metastatic Carcinoma	3027 (0.5)	0.24 (0.2, 0.003–1.0)
AIDS/HIV	1577 (0.3)	0.25 (0.3, 0.003–1.0)
Congestive Heart Failure	11,601 (2.0)	0.35 (0.3, 0.003–1.0)
Peripheral Vascular Disease	13,673 (2.4)	0.32 (0.3, 0.003–1.0)
Cerebrovascular Disease	15,715 (2.7)	0.33 (0.3, 0.003–1.0)
Dementia	11,029 (0.2)	0.39 (0.3, 0.003–1.0)
Chronic Pulmonary Disease	65,379 (11.4)	0.23 (0.3, 0.003–1.0)
Connective Tissue Disease-Rheumatic Disease	10,042 (1.8)	0.26 (0.3, 0.003–1.0)
Peptic Ulcer Disease	3571 (0.6)	0.27 (0.2, 0.003–1.0)
Mild Liver Disease	19,219 (3.4)	0.24 (0.2, 0.003–1.0)

^1^ Statistically significant difference between mean PDC between patients without any diagnoses from the Charlson Comorbidity Index compared to patients with ≥diagnosis (*p* < 0.001).

## Data Availability

Data are held under license by the Skaggs School of Pharmacy and Pharmaceutical Sciences and are not able to be shared.

## Supplementary Materials

The following supporting information can be downloaded at: https://www.mdpi.com/article/10.3390/jpm13111574/s1, Table S1: Number (%) of incident users and average proportion of days covered (PDC) for CPIC Level A, A/B and B medications, stratified by CPIC level.
