# Supplementary material for: Patient-Level Exposure to Actionable Pharmacogenomic Medications in a Nationally Representative Insurance Claims Database
Source: J Pers Med. 2023 Nov 3;13(11):1574. doi: 10.3390/jpm13111574 (PMC10672722; doi:10.3390/jpm13111574)
Supplement: Supplementary file 1 [file jpm-13-01574-s001.zip › jpm-2676244-supplementary.pdf]

Table S1. Number (%) of incident users and average proportion of days covered (PDC) for CPIC Level A, A/B and B medications, stratified by CPIC level [a, b]

| **CPIC Level A (N=487,800)** | | | **CPIC Level A/B (N=21,059)** | | | **CPIC Level B (N=258,994)** | | |
|---|---|---|---|---|---|---|---|---|
| **Medication (Primary Gene)** | **N (%)** | **Mean PDC** | **Medication (Primary Gene)** | **N (%)** | **Mean PDC** | **Medication (Primary Gene)** | **N (%)** | **Mean PDC** |
| **Ivacaftor (CFTR)** | **5 (<0.01)** | **0.893** | **Tetrabenazine (CYP2D6)** | **10 (<0.01)** | **0.621** | **Mycophenolic Acid (HPRT1)** | **908 (0.1)** | **0.564** |
| **Tamoxifen (CYP2D6)** | **1,424 (0.1)** | **0.666** | Venlafaxine (CYP2D6) | 13,312 (1.3) | 0.458 | Sertraline (CYP2C19) | 27,932 (2.7) | 0.436 |
| **Clopidogrel (CYP2C19)** | **7,826 (0.8)** | **0.645** | Hydralazine (NAT2) | 2,979 (0.3) | 0.456 | Methadone (CYP2B6) | 759 (0.1) | 0.434 |
| **Efavirenz (CYP2B6)** | **151 (<0.01)** | **0.638** | Vortioxetine (CYP2D6) | 772 (0.1) | 0.421 | Dexlansoprazole (CYP2C19) | 3,155 (0.3) | 0.422 |
| **Abacavir (HLA-B)** | **82 (<0.01)** | **0.634** | Divalproex Sodium (POLG) | 4,405 (0.4) | 0.383 | Aripiprazole (CYP2D6) | 4,891 (0.5) | 0.404 |
| **Atorvastatin (SLCO1B1)** | **56,599 (5.5)** | **0.615** | Valproic Acid (POLG) | 176 (<0.01) | 0.287 | Fluvoxamine (CYP2D6) | 599 (0.1) | 0.400 |
| **Tacrolimus (CYP3A5)** | **480 (<0.01)** | **0.594** | Pimozide (CYP2D6) | 9 (<0.01) | 0.264 | Risperidone (CYP2D6) | 3,137 (0.3) | 0.364 |
| **Pravastatin (SLCO1B1)** | **16,827 (1.6)** | **0.562** | | | | Clomipramine (CYP2C19) | 1,154 (0.11) | 0.348 |
| Rosuvastatin (SLCO1B1) | 10,382 (1.0) | 0.553 | | | | Trimipramine (CYP2D6) | 927 (0.09) | 0.329 |
| Simvastatin (SLCO1B1) | 24,182 (2.3) | 0.552 | | | | Dapsone (G6PD) | 775 (0.07) | 0.325 |
| Lovastatin (SLCO1B1) | 3,984 (0.4) | 0.550 | | | | Imipramine (CYP2C19) | 611 (0.06) | 0.320 |
| Allopurinol (HLA-B) | 7,088 (0.7) | 0.547 | | | | Desipramine (CYP2D6) | 553 (0.05) | 0.279 |
| Mercaptopurine (TPMT) | 239 (<0.01) | 0.538 | | | | Doxepin (CYP2C19) | 292 (0.03) | 0.263 |
| Fluvastatin (SLCO1B1) | 205 (<0.01) | 0.522 | | | | *Primaquine (G6PD)* | *292 (0.03)* | *0.065* |
| Atazanavir (UGT1A1) | 63 (<0.01) | 0.516 | | | | *Methylene Blue (G6PD)* | *267 (0.03)* | *0.046* |
| Azathioprine (NUDT15) | 960 (0.1) | 0.514 | | | | *Hydrocodone (CYP2D6)* | *38* ***(<0.01)*** | *0.039* |
| Pitavastatin (SLCO1B1) | 755 (0.1) | 0.474 | | | | *Nitrofurantoin (G6PD)* | *4* ***(<0.01)*** | *0.033* |
| Escitalopram (CYP2C19) | 21,992 (2.1) | 0.454 | | | | Moxifloxacin (G6PD) | 0 (0) | -- |
| Citalopram (CYP2C19) | 23,057 (2.2) | 0.437 | | | | Nalidixic Acid (G6PD) | 0 (0) | -- |
| Warfarin (VKORC1) | 7,969 (0.8) | 0.437 | | | | Norfloxacin (G6PD) | 0 (0) | -- |
| Paroxetine (CYP2D6) | 9,078 (0.9) | 0.406 | | | | Sodium Nitrate (G6PD) | 0 (0) | -- |
| Phenytoin (CYP2C9) | 705 (0.1) | 0.397 | | | | Sulfadiazine (G6PD) | 0 (0) | -- |
| Peginterferon Alfa-2A (IFNL3) | 197 (<0.01) | 0.392 | | | | | | |

| CPIC Level A (N=487,800) | | | CPIC Level A/B (N=21,059) | CPIC Level B (N=258,994) |
|---|---|---|---|---|
| Oxcarbazepine (HLA-B) | 1,834 (0.2) | 0.378 | | |
| Pantoprazole (CYP2C19) | 28,652 (2.8) | 0.364 | | |
| Peginterferon Alfa-2B (IFNL3) | 63 (<0.01) | 0.359 | | |
| Omeprazole (CYP2C19) | 70,496 (6.8) | 0.347 | | |
| Carbamazepine (HLA-B) | 1,590 (0.2) | 0.347 | | |
| Amitriptyline (CYP2C19) | 13,143 (1.3) | 0.320 | | |
| Capecitabine (DPYD) | 417 (<0.01) | 0.311 | | |
| Nortriptyline (CYP2D6) | 5,508 (0.5) | 0.306 | | |
| Atomoxetine (CYP2D6) | 1,608 (0.2) | 0.297 | | |
| Lansoprazole (CYP2C19) | 7,597 (0.7) | 0.290 | | |
| Voriconazole (CYP2C19) | 157 (<0.01) | 0.256 | | |
| Celecoxib (CYP2C9) | 10,061 (1.0) | 0.251 | | |
| Irinotecan (UGT1A1) | 3 (<0.01) | 0.238 | | |
| Meloxicam (CYP2C9) | 58,819 (5.7) | 0.207 | | |
| Piroxicam (CYP2C9) | 1,882 (0.2) | 0.186 | | |
| Amikacin (MT-RNR1) | 12 (<0.01) | 0.126 | | |
| Tobramycin (MT-RNR1) | 17 (<0.01) | 0.112 | | |
| Fluorouracil (DPYD) | 61 (<0.01) | 0.098 | | |
| Flurbiprofen (CYP2C9) | 1,014 (0.1) | 0.090 | | |
| *Tramadol (CYP2D6)* | *6,123 (0.6)* | *0.089* | | |
| *Streptomycin (MT-RNR1)* | *2 (<0.01)* | *0.070* | | |
| *Ibuprofen (CYP2C9)* | *140,020 (13.5)* | *0.063* | | |
| *Thioguanine (TPMT)* | *5 (<0.01)* | *0.035* | | |
| *Ondansetron (CYP2D6)* | *81,201 (7.8)* | *0.029* | | |

| CPIC Level A (N=487,800) | | | CPIC Level A/B (N=21,059) | CPIC Level B (N=258,994) |
|---|---|---|---|---|
| *Codeine (CYP2D6)* | *106,201 (10.3)* | *0.028* | | |
| Desflurane (CACNA1S) | 0 (0) | -- | | |
| Fosphenytoin (CYP2C9) | 0 (0) | -- | | |
| Gentamicin (MT-RNR1) | 0 (0) | -- | | |
| Kanamycin (MT-RNR1) | 0 (0) | -- | | |
| Sevoflurane (CACNA1S) | 0 (0) | -- | | |
| Streptomycin (MT-RNR1) | 0 (0) | -- | | |

[a] Drug-gene pairs in bold are those with the 10 highest PDCs in the study; drug-gene pairs in italics are those with the 10 lowest PDCs in the study.

[b] CPIC Level A indicates: “Genetic information should be used to change prescribing of [the] affected drug. Preponderance of evidence is high or moderate in favor of changing prescribing. At least one moderate or strong action (change in prescribing) [is] recommended.” CPIC Level A/B indicates: “Preliminary review indicates it is likely that the definitive CPIC level will be either A or B. Full evidence review [is] needed to assess level of evidence, but prescribing actionability is likely. Full review by expert guideline group [is needed] to assign strength of recommendation.” CPIC level B indicates: “Genetic information could be used to change prescribing of the affected drug because alternative therapies/dosing are extremely likely to be as effective and as safe as non-genetically based dosing. Preponderance of evidence is weak with little conflicting data. At least one optional action (change in prescribing) is recommended.” [5]
